# Breaking the shuttle limitation in aqueous halogen batteries toward durable energy storage systems

**DOI:** 10.1093/nsr/nwag459

**Published:** 2026-07-29

**Authors:** Fulong Zhu, Zhehao Zhang, Fanxing Bu, Dongliang Chao

**Affiliations:** Laboratory of Advanced Materials, Aqueous Battery Center, Electron Microscope Center of Fudan University, Shanghai Key Laboratory of Molecular Catalysis and Innovative Materials, Collaborative Innovation Center of Chemistry for Energy Materials, Shanghai Wusong Laboratory of Materials Science, State Key Laboratory of Porous Materials for Separation and Conversion, College of Smart Materials and Future Energy, Fudan University, China; Laboratory of Advanced Materials, Aqueous Battery Center, Electron Microscope Center of Fudan University, Shanghai Key Laboratory of Molecular Catalysis and Innovative Materials, Collaborative Innovation Center of Chemistry for Energy Materials, Shanghai Wusong Laboratory of Materials Science, State Key Laboratory of Porous Materials for Separation and Conversion, College of Smart Materials and Future Energy, Fudan University, China; Laboratory of Advanced Materials, Aqueous Battery Center, Electron Microscope Center of Fudan University, Shanghai Key Laboratory of Molecular Catalysis and Innovative Materials, Collaborative Innovation Center of Chemistry for Energy Materials, Shanghai Wusong Laboratory of Materials Science, State Key Laboratory of Porous Materials for Separation and Conversion, College of Smart Materials and Future Energy, Fudan University, China; Institute of Cultural Heritage Science and Technology, Key Laboratory of Silicate Cultural Relics Conservation, Ministry of Education, Institute for the Conservation of Cultural Heritage, School of Cultural Heritage and Information Management, Shanghai University, China; Laboratory of Advanced Materials, Aqueous Battery Center, Electron Microscope Center of Fudan University, Shanghai Key Laboratory of Molecular Catalysis and Innovative Materials, Collaborative Innovation Center of Chemistry for Energy Materials, Shanghai Wusong Laboratory of Materials Science, State Key Laboratory of Porous Materials for Separation and Conversion, College of Smart Materials and Future Energy, Fudan University, China

Aqueous batteries have garnered significant attention in large-scale energy storage systems (ESSs) due to their safety advantages and low cost. Although the energy density of aqueous systems is constrained by thermodynamic windows, the collaborative progress of electrolyte engineering and material innovation continues to expand their performance boundaries [1]. Halogen cathodes (HCs), represented by bromine and iodine, show promise for constructing high-performance ESSs due to their multi-electron reaction mechanisms, high redox potentials and rich valence state variations (Fig. 1a) [2]. However, the practical application of HCs has long been hindered by challenges such as the intermediate’s dissolution, shuttle effect and sluggish reaction kinetics. The crossover of polybromides/polyiodides exacerbates anode corrosion and accelerates performance degradation (Fig. 1b). Addressing these issues through electrode-structure or electrolyte-design strategies is a key scientific challenge for promoting the application of halogen batteries (HBs).

**Figure 1.**
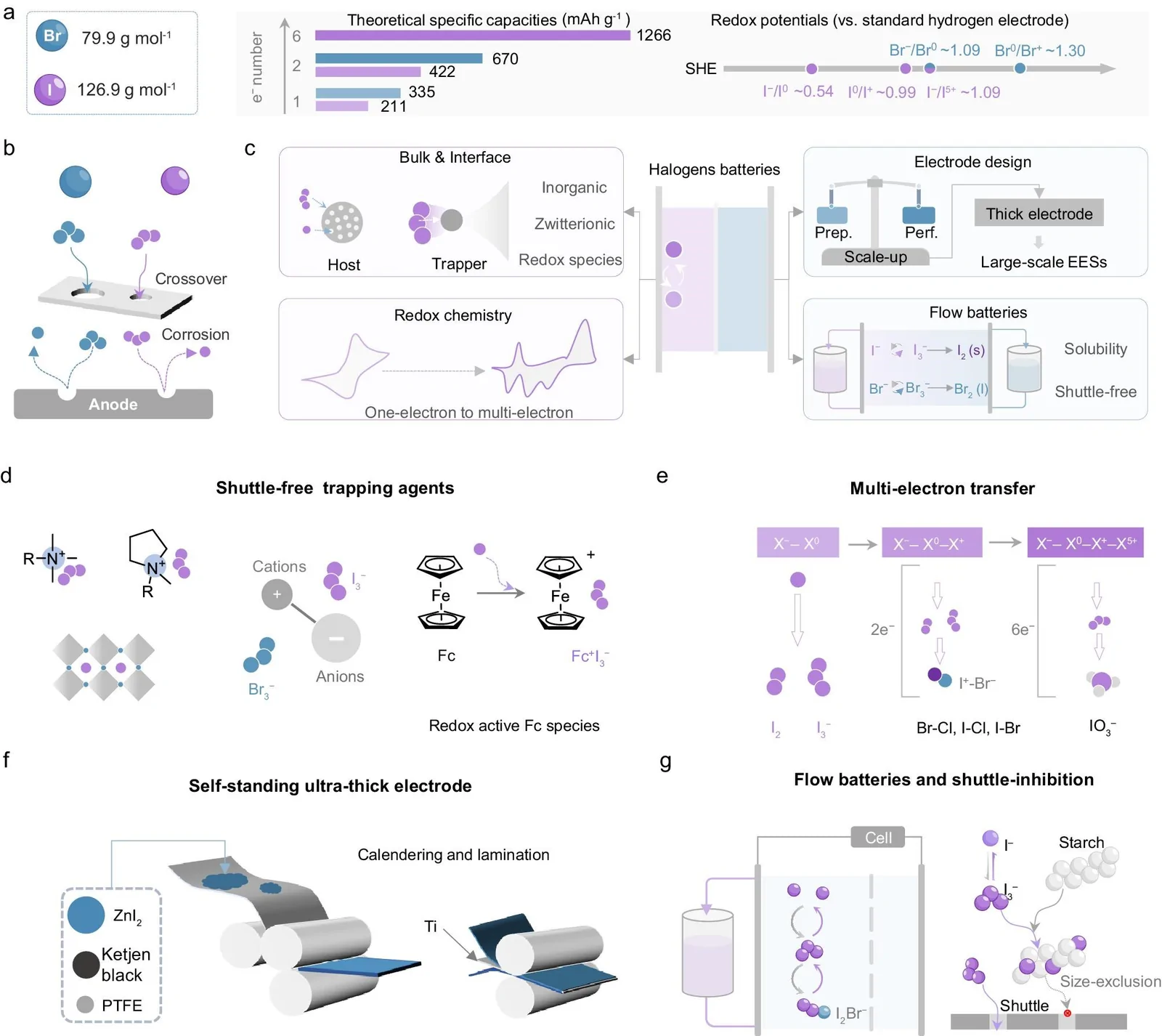
Summary of challenges and multi-strategies toward high-performance aqueous halogen batteries (AHBs). (a) Electrochemical properties of Br and I. (b) Illustration of the two major bottlenecks of the HCs. (c) Multi-strategy synergy from shuttle inhibition to practical application (Prep.: preparation; Perf.: performance). (d) Schematic of trapping agents for shuttle-free AHBs. (e) Illustration of multi-electron transfer halogens: the formation of Br^+^/I^+^, interhalogen compounds and I^5+^ species [4]. (f) Schematic demonstration of the self-standing dry ZnI_2_ electrode [5]. (g) Aqueous halogen flow batteries and shuttle inhibition via starch adsorption.

The research published by Wu *et al*. in *National Science Review* provides a significant breakthrough in addressing the challenges of bromine cathodes. Moving beyond traditional studies that focused on static confinement within the cathode bulk, this work proposes a ‘bulk-interface synergistic regulation’ strategy [3]. In the bulk cathode, cationic sites are introduced through functionalized activated carbon, utilizing microporous electrostatic effects to anchor polybromides and suppress their diffusion driven by concentration gradients. On the interface, 1-butyl-3-methylimidazolium chloride is introduced as an electrolyte additive. Leveraging its spontaneous enrichment along the electric-field direction during discharge, a dynamic ionic barrier layer forms at the interface, actively adsorbing polybromides, promoting their reduction and reuse. This design converts the built-in electric field into a driving force for regulating interfacial behavior, enabling a strategic shift from passive retention to active electric-field guidance. The study elucidates how the internal electric field accelerates the directional migration of polybromides during discharge. It further establishes an electric-field-responsive interface-regulation strategy, addressing the prior neglect of dynamic electric-field effects. Future HB designs should prioritize the dynamic evolution of electrode/electrolyte interfaces and electrochemical mechanisms over the mere pursuit of host materials. Meanwhile, scale-up processes should be integrated with battery system design toward the ultimate development of HBs suitable for large-scale ESSs (Fig. 1c).

The use of trapping agents to enhance the stability of HBs has attracted extensive research interest in recent years (Fig. 1d). The trapping media employed include nitrogen-containing cationic quaternary ammonium salts (e.g. soft-hard zwitterionic trappers, SH-ZITs) [6], non-ionic polymers (e.g. polyacrylamide-I_2_, polyvinylpyrrolidone) and novel inorganic compounds such as halogenated perovskites (e.g. 1,8-octanediamine-SnI_4_) and halogenated MXenes (e.g. Ti_3_C_2_I_2_, Ti_3_C_2_Br_2_) [2]. However, the introduction of non-electrochemically active complexes inevitably reduces the mass proportion of active components in the cathode, thereby limiting improvements in cathode energy density. An iodine-based cathode design strategy employing electrochemically active molecules (e.g. phenazine and ferrocene) as dual-purpose trapping agents achieves an active material mass ratio of up to 88%, effectively inhibiting the shuttle effect while maintaining a high active material loading (Fig. 1d) [7]. This strategy can be further extended to a broader range of HBs.

To further break through the energy density bottleneck of HCs, the potential of multi-electron transfer HBs deserves further exploration (Fig. 1e). A major obstacle lies in the instability of high-valence halogen species (e.g. Br^+^/I^+^) in aqueous media. One effective stabilization strategy is the formation of interhalogen compounds (e.g. BrCl, ICl and IBr) via nucleophilic substitution. Alternatively, nitrogen-containing organic molecules, such as imide groups, can stabilize high-valence iodine through an SN_2_ bimolecular nucleophilic substitution mechanism involving the synergistic cleavage of I–I bonds and nucleophilic attack [8]. Beyond chemical stabilization, suitable catalysts can dramatically accelerate the conversion kinetics, bringing the practical capacity closer to the theoretical value. For instance, mesoporous Zr-UiO-66, featuring abundant interfacial Lewis acid sites, effectively stabilizes the ICl_2_^−^ intermediate and catalyzes the otherwise sluggish I⁺/I_2_ redox, enabling a four-electron zinc–iodine battery with near-theoretical capacity and outstanding cycling stability [9]. Furthermore, the I^−^/IO_3_^−^ redox pair offers a six-electron transfer pathway, fundamentally redefining the theoretical specific capacity ceiling of HBs [4].

Advancing HBs toward practical applications requires consideration of industrial-scale performance metrics. The abundant resources and low cost of halogens provide a material foundation for their large-scale application. However, the performance consistency, scalability and economic viability required for industrial manufacturing go far beyond simply scaling up laboratory research. Extensive electrode design or process optimization strategies have demonstrated engineering feasibility in achieving high areal capacity and constructing Ah-level batteries. Additionally, in terms of manufacturing processes, scalable dry electrode preparation overcomes inherent limitations of traditional iodine-based cathodes, such as low loading, sublimation and poor scalability, achieving an areal capacity exceeding 15.8 mAh cm^−2^ (Fig. 1f) [5]. This process is, in principle, extendable to bromine-based cathode systems. However, the thicker electrode (∼586 μm) significantly extends charge carrier transport pathways. This necessitates in-depth research into the kinetic and thermodynamic characteristics of coupled electron/ion transport at the interface and in the bulk phase to achieve the rate performance required for industrial applications.

Unlike static HCs that require an anchored state, halide ions have also garnered significant attention in flow batteries due to their high solubility. The physical separation of two electrolytes naturally circumvents the shuttle issue of polyhalides (Fig. 1g). However, this introduces phase separation (solubility limitations of elemental halogens) as a core challenge, and the corrosiveness of bromine poses additional engineering challenges that cannot be ignored. In this context, interhalogen compounds overcome the utilization issue of insoluble iodine, while SH-ZITs serve as complexing agents for polyhalides, enabling uniform HBs cycling [6]. To develop more valuable systems, recent years have also seen the development of starch–iodine polymer colloid electrolytes enabling the replacement of expensive Nafion membranes with low-cost porous membranes (Fig. 1g) [10]. In addition, high-valence halogens have also been stably utilized in flow batteries [4]. The development of halogen flow batteries is still in its primary stages, requiring substantial efforts in system development and structural design to accelerate their practical application.

In summary, when the precise regulation of electrochemical conversion mechanisms is coordinated with the systematic optimization of scalable manufacturing processes, coupled with in-depth exploration of multi-electron transfer pathways and the rational design of catalytic mechanisms, HBs are expected to evolve from laboratory-scale candidate products into engineering-practical systems suitable for large-scale ESSs.
